# Plant Disease Control Efficacy of *Platycladus orientalis* and Its Antifungal Compounds

**DOI:** 10.3390/plants10081496

**Published:** 2021-07-21

**Authors:** Sohyun Bae, Jae Woo Han, Quang Le Dang, Hun Kim, Gyung Ja Choi

**Affiliations:** 1 Center for Eco-Friendly New Materials, Korea Research Institute of Chemical Technology, Daejeon 34114, Korea; bshtravel@naver.com; 2 Department of Medicinal Chemistry and Pharmacology, University of Science and Technology, Daejeon 34113, Korea; jaewoo82@krict.re.kr; 3 Research and Development Center of Bioactive Compounds, Vietnam Institute of Industrial Chemistry, Hanoi 100000, Vietnam; ledangquang2011@gmail.com

**Keywords:** *Platycladus orientalis*, terpene, antifungal activity, plant disease control

## Abstract

Plants contain a number of bioactive compounds that exhibit antimicrobial activity, which can be recognized as an important source of agrochemicals for plant disease control. In searching for natural alternatives to synthetic fungicides, we found that a methanol extract of the plant species *Platycladus orientalis* suppressed the disease development of rice blast caused by *Magnaporthe oryzae*. Through a series of chromatography procedures in combination with activity-guided fractionation, we isolated and identified a total of eleven compounds including four labdane-type diterpenes (**1**–**4**), six isopimarane-type diterpenes (**5**–**10**), and one sesquiterpene (**11**). Of the identified compounds, the MIC values of compounds **1**, **2**, **5** & **6** mixture, **9**, and **11** ranged from 100 to 200 μg/mL against *M. oryzae*, whereas the other compounds were over 200 μg/mL. When rice plants were treated with the antifungal compounds, compounds **1**, **2**, and **9** effectively suppressed the development of rice blast at all concentrations tested by over 75% compared to the non-treatment control. In addition, a mixture of compounds **5** & **6** that constituted 66% of the *P. orientalis* ethyl acetate fraction also exhibited a moderate disease control efficacy. Together, our data suggest that the methanol extract of *P. orientalis* including terpenoid compounds has potential as a crop protection agent.

## 1. Introduction

Plant diseases have led to decreased yields and quality seriously hurting agricultural production [1]. The use of synthetic pesticides in modern agriculture has been recognized as one of the most effective control methods for plant diseases, which results in minimizing the losses in yield within an economic threshold [2]. However, the repeated and excessive use of synthetic pesticides over the last few decades has raised concerns for the safety of humans and the destruction of ecosystems and also has led to the emergence of drug-resistant pathogens [3]. Currently, biopesticides have been recognized as one of the most eco-friendly plant disease control methods, and natural resources such as microbes or plant extracts have been used for the development of biopesticides [4].

Plants biosynthesize and accumulate various bioactive substances such as alkaloids, terpenoids, flavonoids, quinones, and phenolic compounds, which can prevent the invasion of pathogens or pests [5]. Moreover, considering that natural compounds isolated from plants are structurally diverse and complex, natural substances can exhibit a broad range of biological activities [6]. Based on antimicrobial activity, many plant extracts and their active compounds have been reported to have antifungal effects in vitro and in vivo [7,8,9,10]. In particular, plant species such as *Azadirachta indica* A. Juss.*, Simmondsia chinensis* (Link) C. K. Schneider, *Reynoutria sachalinensis* F. Schmidt, and *Macleaya cordata* (Wild.) R. Br. have been used commercially as plant protection agents [11].

While searching for plant extracts that are active in the control of plant diseases such as rice blast, tomato gray mold, wheat leaf rust, and pepper anthracnose, we found in this study that the methanol (MeOH) extract of *Platycladus orientalis* exhibits a high disease control efficacy against rice blast. The plant species *P. orientalis* (L.) Franco (=*Thuja orientalis* L.; *Biota orientalis* (L.) Endl.) is an evergreen coniferous tree belonging to the family Cupressaceae [12,13]. Because *P. orientalis* can grow in various climates and soil environments, this plant species is widely distributed all over the world, including India, China, Japan, and Korea [14]. It has been reported that the *P. orientalis* extracts have exhibited various activities such as antioxidant, anticancer, and anti-inflammatory activities [15,16,17], and terpene compounds identified from the *P. orientalis* extracts have shown pharmacological activity [13,18]. However, little is known about the *P. orientalis* extracts and their active compounds against plant diseases caused by fungi.

The current study aimed to identify the antifungal substances from *P. orientalis* extract and to investigate their potential as a biocontrol agent with their in vitro and in vivo antifungal activities. We report here the isolation and identification of 11 compounds based on bioassay-guided fractionation and their in vitro and in vivo antifungal activity against rice blast fungus *Magnaporthe oryzae*. Taken together, our results could provide useful information to develop new eco-friendly crop protecting agents against plant pathogenic fungi.

## 2. Results and Discussion

### 2.1. Discovery of the Platycladus orientalis Showing Plant Disease Control Efficacy

To find the plant extracts exhibiting a plant disease control activity, we performed an in vivo antifungal assay with various plant MeOH extracts and consequently found that the MeOH extract (3000 μg/mL) of *P. orientalis* showed a high disease control efficacy against RCB and WLR both with control values of 90%, but there was no effect on TLB and BPM (Table 1).

For the TGM and PAN, the extract exhibited a moderated control efficacy with control values of 21% and 40%, respectively. When the organic solvent fractions obtained from the *P. orientalis* MeOH extract was investigated for its in vivo antifungal activity, the results showed that the ethyl acetate (EtOAc) fraction exhibited disease control efficacies against RCB and WLR without phytotoxicity, which was comparable to that of the MeOH extract; the EtOAc fraction showed 75%, 21%, and 67% for the control values against RCB, TGM, and WLR at a concentration of 2000 μg/mL, respectively (Table 1). In contrast, the *n*-butanol (BuOH) fraction exclusively exhibited low activities against TGM and WLR, and the water fraction also did not exhibit any in vivo antifungal activities against the tested plant diseases (Table 1). Thus, our results indicate that the EtOAc fraction contains the active compounds exhibiting the antifungal activity against the fungal pathogens causing RCB and WLR, and the causal agent *M. oryzae* for RCB was used for the isolation of active compounds from the EtOAc fraction.

### 2.2. Structural Determination of the Isolated Compounds

**Figure 1:**
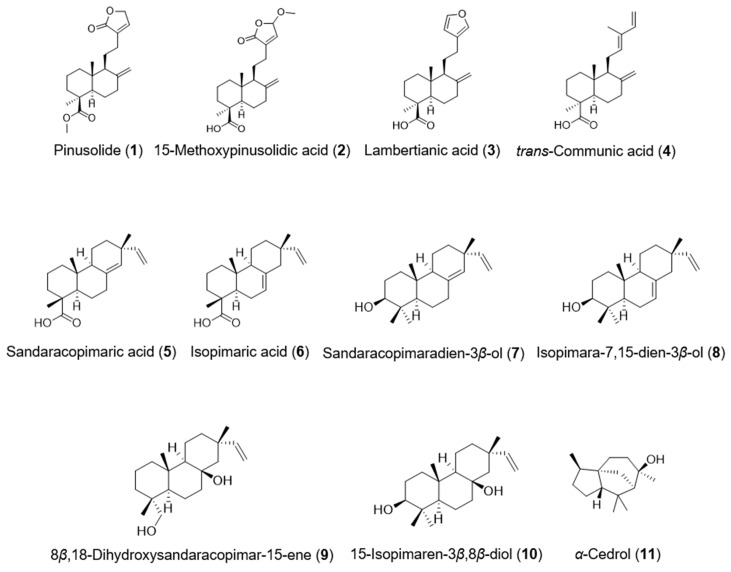
Chemical structures of compounds **1**–**11** isolated from *Platycladus orientalis*.

Bioassay-guided fractionation yielded 11 active constituents identified by nuclear magnetic resonance (NMR) analyses and comparisons with published data: four labdane-type diterpenes (**1**–**4**), six iso pimarane-type diterpenes (**5**–**10**), and one sesquiterpene (**11**) (Figure 1). The constituents were characterized as pinusolide (**1**), 15-methoxypinusolidic acid (**2**), lambertianic acid (**3**), *trans*-communic acid (**4**), sandaracopimaric acid (**5**), isopimaric acid (**6**), sandaracopima radien-3*β*-ol (**7**), isopimara-7,15-dien-3*β*-ol (**8**), 8*β*,18-dihydroxysandaracopimar-15-ene (**9**), 15-isopimaren-3*β*,8*β*-diol (**10**), and *α*-cedrol (**11**). These compounds were identified based on the following evidence (Figures S1–S10).

Compound **1** (pinusolide). ^1^H NMR (CDCl_3_, 500 MHz,): *δ*_H_ 7.09 (1H, *p*, *J* = 1.7 Hz), 4.87 (1H, *d*, *J* = 1.7 Hz), 4.75 (2H, *q*, *J* = 2.0 Hz), 4.56 (1H, *s*), 3.60 (3H, *s*), 2.40 (2H, *m*), 2.15 (1H, *m*), 2.10 (1H, *m*), 1.98 (1H, *m*), 1.93–1.71 (5H, *m*), 1.60 (1H, *m*), 1.52 (2H, *m*), 1.29 (1H, *dd*, *J* = 12.5, 3.1 Hz), 1.17 (3H, *s*), 1.04 (2H, *m*), 0.49 (3H, *s*); ^13^C NMR (CDCl_3_, 125 MHz): *δ*_C_ 177.7, 174.3, 147.4, 143.9, 134.8, 106.6, 70.1, 56.2, 55.6, 51.1, 44.2, 40.2, 39.1, 38.6, 38.1, 28.8, 26.2, 24.6, 21.8, 19.9, 12.5; EIMS m/z 346 [M^+^].

Compound **2** (15-methoxypinusolidic acid).^1^H NMR (CDCl_3_, 500 MHz,): *δ*_H_ 6.75 (1H, *br s*), 5.71 (1H, *br s*), 4.87 (1H, *br s*), 4.54 (1H, *d*, *J* = 6.0 Hz), 3.55 (3H, *s*), 2.45 (1H, *m*), 2.39 (1H, *m*), 2.11 (2H, *m*), 1.96 (1H, *m*), 1.94–1.72 (5H, *m*), 1.60 (2H, *m*), 1.50 (1H, *m*), 1.30 (2H, *m*), 1.22 (3H, *s*), 1.04 (2H, *m*), 0.58 (3H, *s*); ^13^C NMR (CDCl_3_, 125 MHz): *δ*_C_ 183.9, 171.4, 147.2, 141.5, 139.1, 106.8, 102.4, 56.9, 56.1, 55.6, 44.1, 40.4, 39.1, 38.5, 37.8, 28.9, 25.9, 24.5, 21.6, 19.8, 12.7; EIMS m/z 362 [M^+^].

Compound **3** (lambertianic acid). ^1^H NMR (CDCl_3_, 500 MHz,): *δ*_H_ 7.34 (1H, *t*, *J* = 1.7 Hz), 7.19 (1H, *br s*), 6.26 (1H, *br s*), 4.89 (1H, *br s*), 4.57 (1H, *br s*), 3.49 (3H, *s*), 2.56 (1H, *m*), 2.43 (1H, *m*), 2.24 (1H, dt, *J* = 15.2, 7.9 Hz), 2.15 (1H, *m*), 2.01–1.79 (5H, *m*), 1.62 (1H, *m*), 1.23 (3H, *s*), 1.04 (2H, *m*), 0.93 (1H, *m*), 0.80 (1H, *d*, *J* = 7.2 Hz), 0.61 (3H, *s*); ^13^C NMR (CDCl_3_, 125 MHz): *δ*_C_ 182.1, 147.9, 142.7, 138.7, 125.4, 110.9, 106.4, 56.2, 55.2, 50.9, 44.1, 40.4, 39.1, 38.0, 38.7, 29.0, 26.1, 24.2, 23.6, 19.9, 12.8; EIMS m/z 316 [M^+^].

Compound **4** (*trans*-communic acid). ^1^H NMR (CDCl_3_, 500 MHz,): *δ*_H_ 6.32 (1H, *dd*, *J* = 17.4, 10.6 Hz), 5.41 (1H, *m*), 5.03 (1H, *d*, *J* = 17.4 Hz), 4.86 (1H, *d*, *J* = 11.0 Hz), 4.83 (1H, *br s*), 4.45 (1H, *br s*), 2.39 (2H, *m*), 2.20 (1H, *m*), 2.12 (1H, *m*), 1.93 (2H, *m*), 1.88 (1H, *m*), 1.76 (1H, *s*), 1.74 (3H, *s*), 1.53 (2H, *m*), 1.34 (2H, *m*), 1.25 (3H, *s*), 1.14 (1H, *m*), 1.04 (1H, *m*), 0.68 (3H, *s*); ^13^C NMR (CDCl_3_, 125 MHz): *δ*_C_ 182.1, 148.1, 141.5, 134.0, 133.3, 109.8, 107.4, 56.4, 56.2, 44.1, 40.3, 39.3, 38.5, 38.1, 29.1, 25.9, 23.2, 20.0, 12.8, 11.8; EIMS m/z 316 [M^+^].

Compound **5** (sandaracopimaric acid). ^1^H NMR (CDCl_3_, 500 MHz,): *δ*_H_ 5.76 (1H, *dd*, *J* = 17.5, 10.6 Hz), 5.21 (1H, *br s*), 4.95–4.84 (2H, *m*), 2.21 (1H, *dd*, *J* = 14.3, 4.7 Hz), 2.11 (1H, *td*, 13.5, 5.7 Hz), 2.05–1.33 (*m*, 13H), 1.19 (3H, *s*), 1.11 (1H, *m*), 1.03 (3H, *s*), 0.83 (3H, *s*); ^13^C NMR (CDCl_3_, 125 MHz): *δ*_C_ 185.4, 148.9, 136.6, 129.0, 110.1, 50.5, 48.8, 47.3, 38.3, 37.7, 37.4, 37.0, 35.4, 34.4, 26.0, 24.8, 18.5, 18.1, 16.8, 15.2; EIMS m/z 302 [M^+^].

Compound **6** (isopimaric acid). ^1^H NMR (CDCl_3_, 500 MHz,): *δ*_H_ 5.79 (1H, *dd*, *J* = 17.5, 10.7 Hz), 5.32 (1H, *m*), 4.95–4.84 (2H, *m*), 2.05–1.33 (15H, *m*), 1.26 (3H, *s*), 1.11 (1H, *m*), 0.90 (3H, *s*), 0.86 (3H, *s*); ^13^C NMR (CDCl_3_, 125 MHz): *δ*_C_ 185.3, 150.3, 135.6, 121.0, 109.2, 52.0, 46.3, 46.0, 45.0, 38.8, 37.0, 36.8, 36.0, 35.0, 25.1, 21.4, 20.0, 17.9, 17.1, 15.2; EIMS m/z 302 [M^+^].

Compound **7** (sandaracopimaradien-3*β*-ol). ^1^H NMR (CDCl_3_, 500 MHz,): *δ*_H_ 5.80 (1H, *dd*, *J* = 17.4, 10.7 Hz), 5.25 (1H, *m*), 4.93 (2H, *m*), 3.28 (1H, *m*), 2.29 (1H, *m*), 2.06 (1H, *m*), 1.78 (1H, *m*), 1.72–1.26 (11H, *m*), 1.19 (1H, *m*), 1.06 (3H, *s*), 1.03 (3H, *s*), 0.85 (3H, *s*), 0.82 (3H, *s*); ^13^C NMR (CDCl_3_, 125 MHz): *δ*_C_ 149.0, 136.7, 128.9, 110.1, 79.2, 54.2, 50.4, 39.0, 38.1, 37.5, 37.3, 35.9, 34.5, 28.5, 27.6, 26.0, 22.2, 18.8, 15.7, 15.0; EIMS m/z 288 [M^+^].

Compound **8** (isopimara-7,15-dien-3*β*-ol). ^1^H NMR (CDCl_3_, 500 MHz,): *δ*_H_ 5.80 (1H, *dd*, *J* = 17.5, 10.7 Hz), 5.37 (1H, *m*); 4.93 (1H, *dd*, *J* = 17.5, 1.4 Hz), 4.86 (1H, *dd*, *J* = 10.7, 1.4 Hz), 3.26 (1H, *d*, *J* = 10.6 Hz), 1.95 (4H, *m*), 1.86 (1H, *dt*, *J* = 13.3, 3.5 Hz), 1.68–1.51 (3H, *m*), 1.47 (1H, *m*), 1.43–1.29 (3H, *m*), 1.16 (2H, *m*), 0.99 (3H, *s*), 0.90 (3H, *s*), 0.87 (3H, *s*), 0.86 (3H, *s*); ^13^C NMR (CDCl_3_, 125 MHz): *δ*_C_ 150.4, 135.4, 121.5, 109.3, 79.3, 51.9, 50.0, 46.0, 38.6, 37.9, 36.9, 36.1, 35.4, 28.4, 27.4, 23.1, 21.5, 20.1, 15.6, 14.9; EIMS m/z 288 [M^+^].

Compound **9** (8*β*,18-dihydroxysandaracopimar-15-ene). ^1^H NMR (CDCl_3_, 500 MHz,): *δ*_H_ 5.71 (1H, *dd*, *J* = 17.5, 10.7 Hz), 4.85 (1H, *dd*, *J* = 17.5, 1.3 Hz), 4.80 (1H, *dd*, *J* = 10.8, 1.3 Hz), 3.41 (1H, *d*, *J* = 10.9 Hz), 3.09 (1H, *d*, *J* = 10.9 Hz), 1.71 (1H, *m*), 1.67–1.23 (15H, *m*), 1.20 (3H, *s*), 1.02 (3H, *s*), 0.90 (1H, *dd*, *J* = 12.3, 3.0 Hz), 0.84 (1H, *m*), 0.78 (3H, *s*); ^13^C NMR (CDCl_3_, 125 MHz): *δ*_C_ 151.5, 108.6, 72.5, 71.9, 56.8, 51.5, 49.4, 43.1, 38.9, 38.1, 37.6, 37.0, 36.4, 35.3, 24.2, 17.7, 17.5, 17.5, 17.0, 16.0; EIMS m/z 306 [M^+^].

Compound **10** (15-isopimaren-3*β*,8*β*-diol). ^1^H NMR (CDCl_3_, 500 MHz,): *δ*_H_ 5.71 (1H, *dd*, *J* = 17.5, 10.7 Hz), 4.86 (1H, *dd*, *J* = 17.5, 1.3 Hz), 4.81 (1H, *dd*, *J* = 10.7, 1.3 Hz), 3.21 (1H, *dd*, *J* = 11.0, 5.28 Hz), 2.32 (1H, *t*, *J* = 7.5 Hz), 1.75 (1H, *dt, J* = 12.9, 3.6 Hz), 1.70–1.61 (5H, *m*), 1.58 (1H, *m*), 1.53 (1H, *m*), 1.47 (1H, *m*), 1.40–1.28 (4H, *m*), 1.25 (3H, *s*), 1.21 (3H, *s*), 0.98 (3H, *s*), 0.88 (1H, *m*), 0.83 (1H, *m*), 0.80 (3H, *s*); ^13^C NMR (CDCl_3_, 125 MHz): *δ*_C_ 151.5, 108.6, 79.0, 72.4, 56.8, 55.5, 51.4, 43.5, 38.9, 38.0, 37.7, 37.0, 36.5, 28.2, 27.2, 24.2, 17.6, 17.2, 15.7, 15.5; EIMS m/z 306 [M^+^].

Compound **11** (*α*-cedrol). EIMS m/z 222 [M^+^]. **11** was identified as *α*-cedrol by comparing the gas chromatography/mass spectrometry (GC/MS) retention time and mass spectrum to those of an authentic standard of *α*-cedrol (Figure S10). Compounds **5** and **6** were obtained as an inseparable mixture at a ratio of approximately 1:2, which was deduced from the difference in the intensity of the ^1^H-NMR resonances (Figure S5). The ^1^H and ^13^C NMR spectra of **5** and **6** could be individually assigned because their peak intensities were obviously different in the ^1^H and ^13^C NMR spectra.

### 2.3. In Vitro Antifungal Activity of the Identified Compounds

All isolated compounds (**1–11**) were evaluated for their in vitro antifungal activity against five plant pathogenic fungi: *Alternaria brassicicola*, *Botrytis cinerea*, *Colletotrichum coccodes*, *M. oryzae*, and *Phytophthora infestans*. Among these fungi, the causal agent *M. oryzae* for RCB was the species most sensitive to the identified compounds (Table 2), which were comparable to the results of disease control efficacy assay derived from the MeOH extract (Table 1). The minimum inhibitory concentration (MIC) values of pinusolide (**1**), 15-methoxypinusolidic acid (**2**), sandaracopimaric acid (**5**) & isopimaric acid (**6**) mixture, 8*β*,18-dihydroxysandaracopimar-15-ene (**9**), and *α*-cedrol (**11**) were either 100 or 200 μg/mL against *M. oryzae*. For the fungal pathogen *P. infestans,* compounds **2**, **9**, and **11** also exhibited MIC values of 100 μg/mL. For *A. brassicicola*, *B. cinerea*, and *C. coccodes,* the MIC values of all the compounds were over 400 μg/mL (Table 2). Of these active compounds, the pharmaceutical activities of compounds **1**, **2**, and **9** have been reported including their anti-tumor, anti-inflammatory, or neuroprotective activities; but their antimicrobial activity has not been investigated yet [19,20,21]. In this study, we found that the labdane diterpenes **1** and **2** exhibited an antifungal activity for the first time. However, the other labdane diterpenes lambertianic acid (**3**) and *trans*-communic acid (**4**) did not exhibit antifungal activity, suggesting that the lactone moiety linked at C-12 of compounds **1** and **2** is important for their antifungal activity. Isopimaric acid (**6**) from the bark of *Cryptomeria japonica* exhibited 70% and 74% of the inhibitory activity against the mycelial growth of *Rhizoctonia solani* and *M. oryzae* at a concentration of 100 μg/mL, respectively [22]. Compound **11** of the essential oil from *Cunninghamia lanceolata* var. *konishii* exhibited a moderated antifungal activity against several fungi including *Aspergillus niger, Rhizoctonia solani*, and *Fusarium* spp. [23,24], which was similar to our observation in which compound **11** exhibited MIC values of 100 μg/mL against *M. oryzae* and *P. infestans*. Besides the antifungal activity of compound **11**, antibacterial activity has been reported against *Bacillus cereus* and *Staphylococcus epidermidis* both with MIC values of 15.6 μg/mL [24], and antibacterial activity of sandaracopimaradien-3*β*-ol (**7**) has been also reported against *Staphylococcus aureus* [25].

### 2.4. Effects of Compounds **1**, **2**, **9**, and **11** on Fungal Development of Magnaporthe oryzae

**Figure 2:**
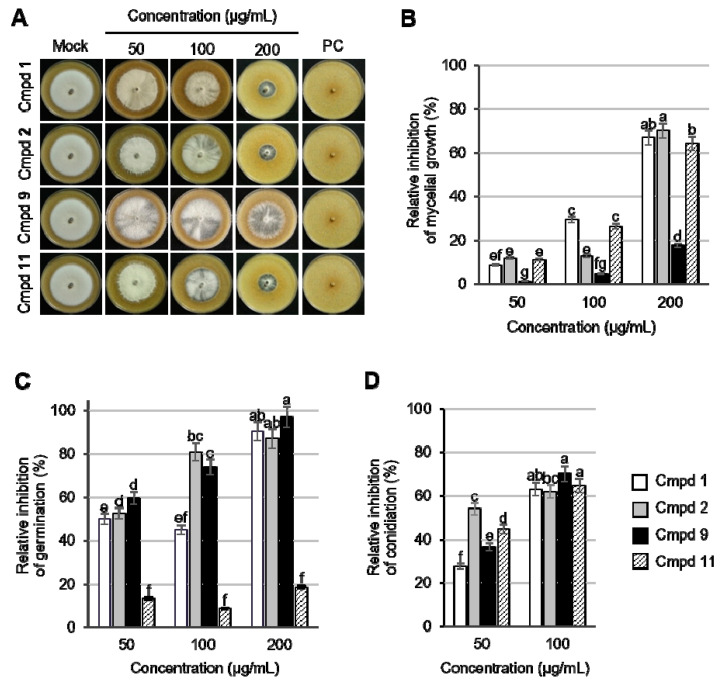
Effects of compounds **1**, **2**, **9**, and **11** on the development of *Magnaporthe oryzae*. (**A**) Mycelial growth of *M. oryzae* grown on RPA medium at 7 dpi. PC, cycloheximide (5 μg/mL) (**B**) Relative inhibition of mycelial growth at 7 dpi. (**C**) Relative inhibition of germination at 6 hpi. (**D**) Relative inhibition of conidiation at 10 dpi. Relative inhibition (%) for all experiments was calculated by comparison to the non-treatment control. The bars represent the mean ± standard deviation of two runs with three replicates. The bars with different letters are significantly different from each other at *p* < 0.01 according to Duncan’s new multiple range test. Cmpd **1**, pinusolide (white bar); cmpd **2**, 15-methoxypinusolidic acid (grey bar); cmpd **9**, 8*β*,18-dihydroxysandaracopimar-15-ene (black bar); cmpd **11**, *α*-cedrol (lined bar).

To explore the effects of the active compounds on the ability of the fungal growth, germination, and conidiation of *M. oryzae,* pinusolide (**1**), 15-methoxypinusolidic acid (**2**), 8*β*,18-dihydroxysandaracopimar-15-ene (**9**), and *α*-cedrol (**11**) that exhibited a promising MIC value (Table 2) were investigated for their inhibitory effects on *M. oryzae* grown on a medium containing different concentrations of each compound. In comparison with the non-treatment control, compounds **1**, **2**, and **11** inhibited mycelial growth of *M. oryzae* ranging from 64% to 70% at a concentration of 200 μg/mL, whereas compound **9** had a growth inhibition rate of 18% at the same concentration (Figure 2A,B). In terms of sesquiterpene, a similar result has been reported for cedrol from *Juniperus virginiana* which exhibited 61% of the inhibitory activity against the mycelial growth of weed decay fungus *Gloeophyllum trabeum* at a concentration of 500 μg/mL [26].

In the germination assay, pinusolide (**1**), 15-methoxypinusolidic acid (**2**), and 8*β*,18-dihydroxysandaracopimar-15-ene **(9**) exhibited an inhibition rate of 91%, 87%, and 97%, at a concentration of 200 μg/mL, respectively, whereas the inhibition rate of *α*-cedrol (**11**) was less than 20% at all the tested concentration (Figure 2C). These results suggest that compounds **1**, **2**, and **9** seemed to be more effective against germination rather than against mycelial growth. In contrast to these compounds, compound **11** was more effective against mycelial growth. When examining the number of *M. oryzae* conidia grown on rice polish agar (RPA) medium containing each compound (50 and 100 μg/mL), compounds **1**, **2**, **9**, and **11** reduced the conidium production ranging from 62% to 70% compared to the non-treatment control at a concentration of 100 μg/mL (Figure 2D). Taken together, the major active compounds **1**, **2**, and **9** are likely to be more effective against germination rather than against mycelial growth. Considering that fungal spore germination is the key process required to initiate vegetative growth and ultimately cause disease [27], compounds **1**, **2**, and **9** can be used for the development of new antifungal agents targeting the fungal spore germination process.

### 2.5. Disease Control Efficacy of the Active Compounds

**Figure 3:**
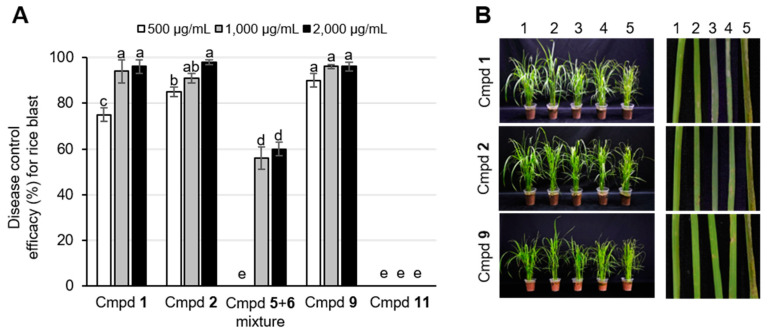
Disease control efficacy of the compounds isolated from *Platycladus orientalis* against rice blast. (**A**) Disease control efficacy for rice blast treated by each compound with different concentrations. The bars represent the mean ± standard deviation of two runs with three replicates. The bars with different letters are significantly different from each other at *p* < 0.01 according to Duncan’s new multiple range test. Cmpd **1**, pinusolide; cmpd **2**, 15-methoxypinusolidic acid; cmpd **5**, sandracopimaric acid; cmpd **6**, isopimaric acid; cmpd **9**, 8*β*,1*8*-dihydroxysandaracopimar-15-ene; cmpd **11**, *α*-cedrol. (**B**) Representative plants and leaves with rice blast disease. Line 1, blasticidin-S (50 µg/mL) treatment as a positive control; line 2, 2000 µg/mL treatment of each compound; line 3, 1000 µg/mL treatment of each compound; line 4, 500 µg/mL treatment of each compound; line 5, 0.025% Tween 20 solution treatment as a negative control.

The rice blast fungus *M. oryzae* infects rice at all stages of growth, resulting in the reduction of the yield and quality of rice [28]. The rice blast is a polycyclic disease spread by asexual spores that infect foliar parts of rice plants by the formation of a special infection structure called appressorium, and then once inside the tissue, the invasive hyphae quickly colonize in the living host cells [28]. Based on our in vitro antifungal activity assay results (Table 2), we selected five active compounds to investigate the in vivo disease control efficacy against RCB: pinusolide (**1**), 15-methoxypinusolidic acid (**2**), sandaracopimaric acid (**5**) & isopimaric acid (**6**) mixture, 8*β*,18-dihydroxysandaracopimar -15-ene (**9**), and *α*-cedrol (**11**). The treatment with compounds **1**, **2**, and **9** (1000 and 2000 μg/mL) exhibited disease control efficacies of over 90% against RCB compared with the non-treatment control (Figure 3).

However, compound **11** had no effect on the disease control on RCB, although compound **11** had the same MIC value as compound **2** (Table 2). At a concentration level of 500 μg/mL, compounds **1**, **2**, and **9** exhibited disease control efficacies of 75%, 85%, and 90%, respectively, against RCB (Figure 3). In addition, the **5** & **6** mixture also exhibited moderate disease control efficacies of 56% and 60% against RCB at a concentration level of 1000 and 2000 μg/mL, respectively. During the disease control efficacy assay, we observed that no phytotoxic symptoms appeared on the compound-treated plants (Figure S11).

It has been reported that rice plants produce pimarane-type diterpene phytoalexins including oryzalexins, momilactones, and phytocassanes when rice plants were infected by *M. oryzae* [29,30,31,32]. These phytoalexins were structurally similar to compounds **5**–**10** identified in this study, and the diterpene phytoalexins of rice also showed the inhibitory effect on spore germination and germ tube growth of *M. oryzae* [32,33,34,35,36]. Although oryzalexin D was proposed to be a detergent-like mechanism with disruption or alteration of cell membrane permeability [36], the detailed mechanisms of antifungal action of other ditepene phytoalexins are still lacking.

Similar to our results, previous investigations showed that an ethanolic crude extract and petroleum ether fractions from *P. orientalis* exhibited a disease control efficacy ranging from 62% to 76% for rice sheath blight caused by *R. solani* at a concentration level of 1000 μg/mL [26]. In contrast to our results, Wang et al. [37] identified two diterpenoids totarol and sclareol as the antifungal compounds from *P. orientalis.* Because totarol and sclareol are only partially responsible for the in vitro and in vivo antifungal activity of the *P. orientalis* extracts, Wang et al. [37] suggested that the antifungal effect exerted by the *P. orientalis* extracts depends on the synergism of many compounds and that a single component from *P. orientalis* has limited functions in the total antifungal activity. However, in this study, we did not observe any synergistic effects between active compounds (**1**, **2**, **9**, and **11**) in the in vitro assays (Table S1).

### 2.6. Constituent Analysis of the Platycladus orientalis EtOAc Fraction

**Figure 4:**
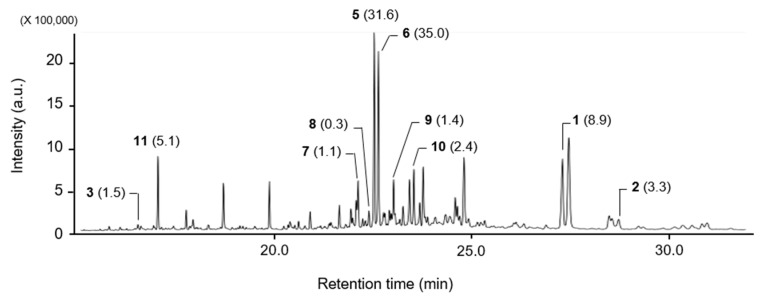
Constituent analysis of the *Platycladus orientalis* EtOAc fraction. Parentheses indicate relative peak area (%) of each compound. **1**, pinusolide; **2**, 15-methoxypinusolidic acid; **3**, lambertianic acid; **5**, sandracopimaric acid; **6**, isopimaric acid; **7**, sandaracopimaradien-3*β*-ol; **8**, isopimara-7,15-dien-3*β*-ol; **9**, 8*β*,18-dihydroxysandaracopimar-15-ene; **10**, 15-isopimaren-3*β*,8*β*-diol; **11**, *α*-cedrol.

For the quantification of the identified compounds in the *P. orientalis* EtOAc fraction, quantitative analysis was performed with GC/MS. Of the 11 identified compounds, the major constituents of the *P. orientalis* EtOAc fraction consisted of sandaracopimaric acid (**5**) and isopimaric acid (**6**); 31.6% and 35% of the EtOAc fraction consisted of compounds **5** and **6,** respectively, (Figure 4).

Considering the compositional ratio and the MIC values of the **5** and **6** mixture, compounds **5** and **6** might contribute to the activity of the *P. orientalis* MeOH extract and EtOAc fraction. However, **5** and **6** did show a moderate in vivo antifungal activity against rice blast. Given that the composition ratio of pinusolide (**1**) and 15-methoxypinusolidic acid (**2**) was 8.9% and 3.3%, respectively, compounds **1** and **2** might be one of the major compounds of the *P. orientalis* extract in terms of antifungal activity because both **1** and **2** showed a promising in vitro and in vivo antifungal activity. In terms of quantity, the fourth most abundant compound was *α*-cedrol (**11**; 5.1%); however, it had relatively little effect on the activity of the MeOH extract and EtOAc fraction because **11** exhibited no disease control efficacy. In addition, although 8*β*,18-dihydroxysandaracopimar-15-ene (**9**) exhibited the strongest in vivo antifungal activity against rice blast, compound **9** accounted for a small portion (1.4%) of the EtOAc fraction, suggesting that it has relatively little effect on the activity of the MeOH extract and EtOAc fraction.

## 3. Materials and Methods

### 3.1. Plant Material and Fungal Strains

The leaves and stems of *P. orientalis* were collected by the Research and Development Center of Bioactive Compounds at the Vietnam Institute of Industrial Chemistry (VIIC, Hanoi, Vietnam), and voucher specimens were identified by Dr. Tran Bach at the Institute of Ecology and Biological Resources (Hanoi, Vietnam) and deposited in the laboratory of VIIC. The collected plant materials were air-dried and finely macerated by a blender for further study. The powder material of *P. orientalis* (15 kg) was extracted in MeOH at room temperature for 24 h without shaking, and then, the extracts were filtered through filter paper (Whatman No. 1; Merck, Kenilworth, NJ, USA). The filtrates were concentrated at 40 °C by a rotary evaporator (Rotavapor R-300; Büchi, Flawil, Switzerland), yielding 100 g of a MeOH extract.

For the antifungal activity assay, we used five phytopathogenic fungi *A. brassicicola* KACC 40036, *B. cinerea* KACC 48736, *C. coccodes* KACC 48737, *M. oryzae* KACC 46552, and *P. infestans* KACC 48738, which were provided by the Korean Agricultural Culture Collection (KACC, Jeonju, Korea). Fungal sporulation and maintenance were performed as described previously [38,39]. Additionally, we used two obligate parasitic fungi *Puccinia triticina* for wheat leaf rust and *Blumeria graminis* f. sp. *hordei* for barley powdery mildew, which were maintained on their hosts.

### 3.2. Isolation of Antifungal Compounds from Platycladus orientalis

A procedure for the isolation of the active compounds is shown in a flowchart (Figure S12). The whole (100 g) dry MeOH extract was suspended in 5 L of water and successively partitioned twice with the same amount of EtOAc and BuOH, sequentially. Each fraction was concentrated to dryness under reduced pressure using a Rotavapor R-300 rotary evaporator (Büchi) at 40 °C to yield an EtOAc fraction (46 g), BuOH fraction (35 g), and water fraction (18 g). Because the EtOAc fraction exclusively had effects on the disease development of rice blast and wheat leaf rust, the EtOAc fraction was used to identify the active components using various chromatographic processes [8,9,10]. The EtOAc fraction was loaded onto a silica gel column (70–230 mesh; Merck, Darmstadt, Germany) and successively eluted with a gradient of hexane/EtOAc (95:5 to 0:100, *v*/*v*), yielding twelve fractions E1–E12. Based on the in vitro antifungal activity against *M. oryzae*, four fractions E2 (6.4 g), E3 (3 g), E5 (3 g), and E7 (2 g) were selected as active fractions containing antifungal compounds.

The fraction E2 was separated onto a silica gel column (70–230 mesh; Merck) using a gradient of hexane/dichloromethane (DCM) (8:2 to 0:10, v/v) to yield nine fractions E21–E29. Compounds **3** (45 mg) and **4** (95 mg) were finally purified from the active fraction E26 (730 mg) using a LC-6AD high-performance liquid chromatography (HPLC) system (Shimadzu, Kyoto, Japan) equipped with a Capcell Pak C18 UG column (20 × 250 mm, 5 μm; Phenomenex, Torrance, CA, USA). The column was eluted with a linear gradient (85–100%) of aqueous MeOH at a flow rate of 5 mL/min, and the effluent was monitored with the SPD-M10Avp photodiode array detector (Shimadzu). Compound **11** (108 mg) was purified from the fraction E27 (432 mg), which was dissolved in 1 mL petroleum ether and crystallized at −4 °C for 4 h. The active fraction E3 was further purified by a medium-pressure liquid chromatography (MPLC) system (Isolera One; Biotage, Uppsala, Sweden) equipped with a SNAP KP-Sil 25 g cartridge (Biotage) and then eluted with a gradient of hexane/DCM (9:1 to 0:10, *v*/*v*) to yield the pure compounds **7** (488 mg) and **8** (207 mg). The active fraction E5 was separated using an MPLC system (Biotage) equipped with a SNAP KP-silica column (Biotage) with a gradient of DCM/EtOAc (10:0 to 9:1, *v*/*v*) at a flow rate of 5 mL/min, yielding five fractions E51–E55. The active faction E52 was pure compound **1** (815 mg), and the fraction E54 was further purified on a SNAP KP-silica column of an MPLC system with a gradient of hexane/EtOAc (93:7 to 8:2, *v*/*v*) at a flow rate of 3 mL/min, giving two pure compounds **9** (43 mg) and **10** (40 mg). Compound **2** (450 mg) was purified from the active fraction E7 by a ZIP Sphere silica gel column (Biotage) eluted with a gradient of hexane/DCM (97:3 to 88:12, *v*/*v*) at a flow rate of 5 mL/min. In addition, the mixture of compounds **5** and **6** (580 mg) was isolated from the active fractions E28 and E3 with the MPLC system (Biotage). All solvents used for the chromatography analyses were purchased from Samchun Pure Chemical Co., Ltd. (Seoul, Korea) and used without further purification.

### 3.3. General Experimental Procedures for Chemical Analysis

The chemical structures of the pure compounds isolated from *P. orientalis* extract were determined by spectroscopic analyses and compared with the spectroscopic data of compounds previously described in the literatures [19,21,22,40,41,42,43,44,45,46]. The NMR experiments were carried out on a Bruker Advance Spectrometer (Burker BioSpin, Rheinstetten, Germany) at 500 MHz for proton(^1^H) and 125 MHz for carbon-13 (^13^C) in CDCl_3_ (Cambridge Isotope Laboratories, Tewksbury, MA, USA). Chemical shifts were referenced to the solvent peaks (δ_C_ 77.0 and δ_H_ 7.26 for CDCl_3_)

### 3.4. Determination of the Minimum Inhibitory Concentration

To determine the MIC values of the isolated compounds against phytopathogenic fungus, the broth microdilution assay was performed using two-fold serial dilutions [47]. Briefly, each purified compound was serially diluted by transferring 200 μg/mL into the wells of 96-well microtiter plates that contain a spore suspension (1 × 10^5^ spores/mL) of fungal pathogens. As a positive and negative control, we used the chemical fungicide blasticidin-S and potato dextrose broth (PDB) medium containing 1% dimethyl sulfoxide (DMSO), respectively. The microtiter plates were incubated for 2 days, and the MIC values were determined by visual inspection of complete growth inhibition. The assay was performed two times with three replicates

### 3.5. Inhibition Assay for Mycelial Growth, Conidiation, and Germination

To investigate the effects of the active compounds on mycelial growth, an agar plug of *M. oryzae* culture was inoculated onto RPA (32 g rice polish, 10 g dextrose, 12 g agar and 1 L distilled water) medium that was prepared by adding the compounds at a final concentration of 50, 100, and 200 μg/mL in DMSO. The colony diameter of each culture plate was measured at 7 days post-inoculation/incubation (dpi). Effect of the active compounds on conidiation was evaluated at 10 dpi by counting the number of conidia harvested with 2–3 mL of sterilized distilled water from 10-day-old RPA culture plates containing different concentrations of active compounds. To explore the inhibitory effects of the active compounds on germination, 70 μL of conidial suspension (1 × 10^5^ conidia/mL) in PDB containing different concentrations of active compounds were placed onto a glass slide and incubated in a moist container at 25 °C. The germination rates at 6 h post- inoculation/incubation (hpi) were calculated by microscopic observation, and more than 100 conidia from each treatment were examined in each experiment. All experiments were performed with three replications and two repetitions. The relative inhibition (%) of mycelial growth, germination, and conidiation was determined by comparison with a non-treated control.

### 3.6. Disease Control Efficacy Assay

To explore the effects of the extracts and pure compounds on the control of plant diseases, the solvent extracts (2000 and 3000 μg/mL) and pure compounds (1000 μg/mL) were prepared by dissolving in a 5% aqueous MeOH solution. Chemical fungicides and 5% aqueous MeOH including 0.025% Tween 20 were used as a positive and negative control, respectively. Treatment of each compound onto plants was performed one day before the inoculation of the pathogen. The tested plant diseases were as follows: rice blast (RCB; caused by *M. oryzae*), tomato late blight (TLB; caused by *P. infestans*), tomato gray mold (TGM; caused by *B. cinerea*), wheat leaf rust (WLR; caused by *P. triticina*), barley powdery mildew (BPM; caused by *B. graminis* f. sp. *hordei*), and pepper anthracnose (PAN; caused by *C. coccodes*). The preparation of the plants, inoculation method, and evaluation of the control efficacy were performed as previously described [10]. Briefly, for the development of RCB, two or three-leaf stages of rice plants were inoculated by spraying with a spore suspension (5 × 10^5^ conidia/mL) of *M. oryzae*. The inoculated plants were incubated at 25 °C for 5 days, and then the disease control efficacy was calculated with the following equation: control efficacy (%) = 100 × (1 − B/A), where A is the mean of lesion area (%) on the leaves of the control plants, and B is the mean of lesion area (%) on the leaves of the treated plants [10]. Developmental conditions for other plant diseases were described in Table S2. All experiments were conducted twice with three replicates for each treatment.

### 3.7. Relative Quantification of the Isolated Compounds from Platycladus orientalis EtOAc Fraction

To investigate the compositional ratio of the isolated compounds present in the *P. orientalis* EtOAc fraction, we performed GC/MS analysis. Briefly, the EtOAc fraction (100 μg) was mixed with 30 μL of methoxyamine hydrochloride solution (20 mg/mL in pyridine), 50 μL of *N*,*O*-bis(trimethylsilyl)trifluoroacetamide solution containing 1% trimethylchlorosilane, and 10 μL of 2-chloronaphthalene (250 μg/mL in pyridine). The samples were incubated for 1 h at 60 °C for derivatization, and then, the resulting products were directly analyzed using a GCMS-QP2020 (Shimadzu) including a Restek Rxi-5MS column (30 m × 0.25 mm, ID 0.25 μm film thickness; Bellefonte, PE, USA), with helium as the carrier gas at a constant linear velocity of 47.2 cm/s. The GC inlet temperature was set to 250 °C. The column temperature was increased from 80 to 260 °C at a rate of 10 °C/min, and the holding time was 10 min at 260 °C. The interface and ion source temperatures were maintained at 280 and 230 °C, respectively. Relative quantification was performed based on the relative peak area (%) of each compound.

### 3.8. Statistical Analysis

Statistical analysis was performed by using R software packages (version 4.0.5). All of the analyses were performed in triplicate with two runs and expressed as mean ± standard deviation. Differences were tested with one-way analysis of variance (ANOVA) followed by Duncan’s new multiple range test (*p* < 0.01).

## 4. Conclusions

In the current study, we found that the MeOH extract of the plant *P. orientalis* effectively suppressed the disease development of rice blast caused by *M. oryzae*. Through a series of chromatography procedures in combination with activity-guided fractionation, we identified a total of eleven compounds including four labdane-type diterpenes (**1**–**4**), six isopimarane-type diterpenes (**5**–**10**), and one sesquiterpene (**11**). When rice plants were treated with compounds **1**, **2**, **9**, **5** & **6** mixture, and **11**, compounds **1**, **2**, and **9** suppressed the development of rice blast by over 75% compared to the non-treatment control at all concentrations tested. It is the first time that the in vitro and in vivo antifungal activities of compounds **1**, **2**, and **9** are reported in terms of their biological activity. Based on in vitro and in vivo antifungal activities, our results suggest that the MeOH extract of *P. orientalis* including the terpenoid compounds has potential as a crop protection agent for rice blast.

## 5. Patents

Results from the work reported in this manuscript were patented (Korea Patent Registration No. 1022409720000).

## Figures and Tables

**Table 1:** Plant disease control efficacy of the *Platycladus orientalis* MeOH extract and its partitioned fractions.

Treatment	Concentration (µg/mL)	Disease Control Efficacy (%)					
		RCB	TGM	TLB	WLR	BPM	PAN
MeOH extract	3000	90 ± 2 ^ab^	21 ± 3 ^b^	0 ^b^	90 ± 5 ^a^	0 ^b^	40 ± 1 ^c^
EtOAc fraction	2000	75 ± 5 ^b^	21 ± 3 ^b^	0 ^b^	67 ± 7 ^b^	0 ^b^	0 ^d^
BuOH fraction	2000	0 ^c^	14 ± 5 ^bc^	0 ^b^	20 ± 3 ^c^	0 ^b^	0 ^d^
Water fraction	2000	0 ^c^	0 ^c^	0 ^b^	0 ^d^	0 ^b^	0 ^d^
Blasticidin-S	1	82 ± 2 ^b^	−	−	−	−	−
	50	100 ^a^	−	−	−	−	−
Fludioxonil	5	−	85 ± 4 ^a^	−	−	−	−
	50	−	100 ^a^	−	−	−	−
Dimethomorph	2	−	−	92 ± 3 ^a^	−	−	−
	10	−	−	100 ^a^	−	−	−
Flusilazole	2	−	−	−	84 ± 2 ^ab^	92 ± 5 ^a^	−
	10	−	−	−	100 ^a^	100 ^a^	−
Dithianon	10	−	−	−	−	−	75 ± 5 ^b^
	50	−	−	−	−	−	96 ± 2 ^a^

The values represent the mean ± standard deviation of two runs with three replicates. Values with different letters are significantly different at *p* < 0.01 according to Duncan’s multiple range test. RCB, rice blast; TGM, tomato gray mold; TLB, tomato late blight; WLR, wheat leaf rust; BPM, barley powdery mildew; and PAN, pepper anthracnose. –, not tested.

**Table 2:** In vitro antifungal activity of the compounds isolated from *Platycladus orientalis*.

Phytopathogenic Fungus	MIC (μg/mL) of Compound									
	1	2	3	4	5 & 6	7	8	9	10	11
*Alternaria brassicicola*	−	−	−	−	−	−	−	−	−	−
*Botrytis cinerea*	−	−	−	−	−	−	−	−	−	−
*Colletotrichum coccodes*	−	−	−	−	−	−	−	−	−	−
*Magnaporthe oryzae*	100	200	−	−	100	−	−	100	−	200
*Phytophthora infestans*	−	100	−	−	−	−	−	100	−	100

**1**, pinusolide; **2**, 15-methoxypinusolidic acid; **3**, lambertianic acid; **4**, *trans*-communic acid; **5**, sandracopimaric acid; **6**, isopimaric acid; **7**, sandaracopimaradien-3*β*-ol; **8**, isopimara-7,15-dien-3*β*-ol; **9**, 8*β*,18-dihydroxysandaracopimar-15-ene; **10**, 15-isopimaren-3*β*,8*β*-diol; **11**, *α*-cedrol. −, no inhibition at 200 μg/mL.

## Data Availability

Data are contained within the article and supplementary materials.

## Supplementary Materials

The following are available online at https://www.mdpi.com/article/10.3390/plants10081496/s1, Figures S1–S10: NMR spectral data and MS spectrum of compounds **1**–**11**, Figure S11: Plants treated with compounds for phytotoxicity, Figure S12: Isolation scheme of compounds **1**–**11** from the *Platycladus orientalis* extract, Table S1: MIC values against *Magnaporthe oryzae* according to the 1:1 mixing ratio of pure compounds, Table S2: Inoculation and incubation method for plant disease.

## References

[1] Lucas J.A., Barling D. (2017). Fungi, Food Crops, and Biosecurity: Advances and Challenges. Advances in Food Security and Sustainability.

[2] Bandara A.Y., Weerasooriya D.K., Conley S.P., Bradley C.A., Allen T.W., Esker P.D. (2020). Modeling the relationship between estimated fungicide use and disease-associated yield losses of soybean in the United States I: Foliar fungicides vs foliar diseases. PLoS ONE.

[3] Mikaberidze A., Paveley N., Bonhoeffer S., van den Bosch F. (2017). Emergence of resistance to fungicides: The role of fungicide dose. Phytopathology.

[4] Kim B.S., Hwang B.K. (2007). Microbial fungicides in the control of plant diseases. J. Phytopathol..

[5] Molyneux R.J., Lee S.T., Gardner D.R., Panter K.E., James L.F. (2007). Phytochemicals: The good, the bad and the ugly?. Phytochemistry.

[6] Cheng S.S., Chung M.J., Lin C.Y., Wang Y.N., Chang S.T. (2012). Phytochemicals from *Cunninghamia konishii* Hayata act as antifungal agents. J. Agric. Food Chem..

[7] Han J.W., Shim S.H., Jang K.S., Choi Y.H., Kim H., Choi G.J. (2018). In vivo disease control efficacy of isoquinoline alkaloids isolated from *Corydalis ternata* against wheat leaf rust and pepper anthracnose. J. Microbiol. Biotechnol..

[8] Kim B., Han J.W., Ngo M.T., Dnag Q.L., Kim J.-C., Kim H., Choi G.J. (2018). Identification of novel compounds, oleanane- and ursane-type triterpene glycosides, from *Trevesia palmata*: Their biocontrol activity against phytopathogenic fungi. Sci. Rep..

[9] Ngo M.T., Han J.W., Yoon S., Bae S., Kim S.-Y., Kim H., Choi G.J. (2019). Discovery of new triterpenoid saponins isolated from *Maesa japonica* with antifungal activity against rice blast fungus *Magnaporthe oryzae*. J. Agric. Food Chem..

[10] Ngo M.T., Han J.W., Nguyen M.V., Dang Q.L., Kim H., Choi G.J. (2021). Antifungal properties of natural products from *Pterocarya tonkinensis* against phytopathogenic fungi. Pest Manag. Sci..

[11] Kimberly D.G., Rahman A. (2018). Bioactive natural products in plant disease control. Studies in Natural Products Chemistry.

[12] Koo K.A., Sung S.H., Kim Y.C. (2002). A new neuroprotective pinusolide derivative from the leaves of *Biota orientalis*. Chem. Pharm. Bull..

[13] Kim C.S., Choi S.U., Lee K.R. (2012). Three new diterpenoids from the leaves of *Thuja orientalis*. Planta Med..

[14] Shan M.-Q., Shang J., Ding A.-W. (2014). *Platycladus orientalis* leaves: A systemic review on botany, phytochemistry and pharmacology. Am. J. Chin. Med..

[15] Emami S.A., Sadeghi-aliabadi H., Saeidi M., Jafarian A. (2005). Cytotoxic evaluations of Iranian conifers on cancer cells. Pharm. Biol..

[16] Loizzo M.R., Saab A.M., Tundis R., Statti G.A., Menichini F., Lampronti I., Gambari R., Cinatl J., Doerr H.W. (2008). Phytochemical analysis and *in vitro* antiviral activities of the essential oils of seven Lebanon species. Chem. Biodivers..

[17] Srivastava P., Kumar P., Singh D., Singh V. (2012). Biological properties of *Thuja orientalis* Linn. Adv. Life Sci..

[18] Koo K.A., Kim S.H., Lee M.K., Kim Y.C. (2006). 15-Methoxypinusolidic acid from *Biota orientalis* attenuates glutamate-induced neurotoxicity in primary cultured rat cortical cells. Toxicol. In Vitro.

[19] Kim C.S., Suh W.S., Choi S.U., Kim K.H., Lee K.R. (2014). Two new diterpenoids from *Thuja orientalis* and their cytotoxicity. Bull. Korean Chem. Soc..

[20] Koo K.A., Lee M.K., Kim S.H., Jeong E.J., Kim S.Y., Oh T.H., Kim Y.C. (2007). Pinusolide and 15-methoxypinusolidic acid attenuate the neurotoxic effect of staurosporine in primary cultures of rat cortical cells. Br. J. Pharmacol..

[21] Yang H.O., Suh D.Y., Han B.H. (1995). Isolation and characterization of platelet-activating factor receptor binding antagonists from *Biota orientalis*. Planta Med..

[22] Kofujita H., Fujino Y., Ota M., Takahashi K. (2006). Antifungal diterpenes from the bark of *Cryptomeria japonica* D. Don. Holzforschung.

[23] Cheng S.S., Lin C.Y., Gu H.J., Chang S.T. (2011). Antifungal activities and chemical composition of wood and leaf essential oils from *Cunninghamia konishii*. J. Wood Chem. Technol..

[24] Su Y.C., Hsu K.P., Wang E.I.C., Ho C.L. (2012). Composition, anticancer, and antimicrobial activities in vitro of the heartwood essential oil of *Cunninghamia lanceolata* var. *konishii* from Taiwan. Nat. Prod. Commun..

[25] Smith E., Williamson E., Zloh M., Gibbons S. (2005). Isopimaric acid from *Pinus nigra* shows activity against multidrug-resistant and EMRSA strains of *Staphylococcus aureus*. Phytother. Res..

[26] Mun S.P., Prewitt L. (2011). Antifungal activity of organic extracts from *Juniperus virginiana* Heartwood against wood decay fungi. For. Prod. J..

[27] Ortiz S.C., Huang M., Hull C.M. (2019). Spore germination as a target for antifungal therapeutics. Antimicrob. Agents Chemother..

[28] Asibi A.E., Chai Q., Coulter J.A. (2019). Rice blast: A disease with implications for global food security. Agronomy.

[29] Otomo K., Kanno Y., Motegi A., Kenmoku H., Yamane H., Mitsuhashi W., Oikawa H., Toshima H., Itoh H., Matsuoka M. (2004). Diterpene cyclases responsible for the biosynthesis of phytoalexins, momilactones A, B, and oryzalexins A–F in rice. Biosci. Biotechnol. Biochem..

[30] Peters R.J. (2006). Uncovering the complex metabolic network underlying diterpenoid phytoalexin biosynthesis in rice and other cereal crop plants. Phytochemistry.

[31] Toyomasu T., Kagahara T., Okada K., Koga J., Hasegawa M., Mitsuhashi W., Sassa T., Yamane H. (2008). Diterpene phytoalexins are biosynthesized in and exuded from the roots of rice seedlings. Biosci. Biotechnol. Biochem..

[32] Zhao M., Cheng J., Guo B., Duan J., Che C.T. (2018). Momilactone and related diterpenoids as potential agricultural chemicals. J. Agric. Food Chem..

[33] Akatsuka T., Kodama O., Sekido H., Kono Y., Takeuchi S. (1985). Novel phytoalexins (oryzalexins A, B and C) isolated from rice blast leaves infected with *Pyricularia oryzae*. Part I: Isolation, characterization and biological activities of oryzalexins. Agric. Biol. Chem..

[34] Cartwright D., Langcake P., Pryce R.J., Leworthy D.P., Ride J.P. (1977). Chemical activation of host defence mechanisms as a basis for crop protection. Nature.

[35] Koga J., Shimura M., Oshima K., Ogawa N., Yamauchi T., Ogasawara N. (1995). Phytocassanes A, B, C and D, novel diterpene phytoalexins from rice, *Oryza sativa* L.. Tetrahedron.

[36] Sekido H., Akatsuka T. (1987). Mode of action of oryzalexin D against *Pyricularia oryzae*. Agric. Biol. Chem..

[37] Wang H., Wang J., Peng X., Zhou P., Bai N., Meng J., Deng X. (2014). Control efficacy against rice sheath blight of *Platycladus orientalis* extract and its antifungal active compounds. Eur. J. Plant Pathol..

[38] Choi G.J., Jang K.-S., Kim J.-S., Lee S.-W., Cho J.-Y., Cho K.-Y., Kim J.-C. (2004). *In vivo* antifungal activities of 57 plant extracts against six plant pathogenic fungi. Plant Pathol. J..

[39] Choi N.H., Jang J.Y., Choi G.J., Choi Y.H., Jang K.S., Min B.S., Dang Q.L., Kim J.-C. (2017). Antifungal activity of sterols and dipsacus saponins isolated from *Dipsacus asper* roots against phytopathogenic fungi. Pestic. Biochem. Physiol..

[40] Asili J., Lambert M., Ziegler H.L., Staerk D., Sairafianpour M., Witt M., Asghari G., Ibrahimi I.S., Jaroszewski J.W. (2004). Labdanes and isopimaranes from *Platycladus orientalis* and their effects on erythrocyte membrane and on *Plasmodium falciparum* growth in the erythrocyte host cells. J. Nat. Prod..

[41] Block S., Baccelli C., Tinant B., Van Meervelt L., Rozenberg R., Habib Jiwan J.L., Llabrès G., De Pauw-Gillet M.C., Quetin-Leclercq J. (2004). Diterpenes from the leaves of *Croton zambesicus*. Phytochemistry.

[42] Chien S.C., Liu H.K., Kuo Y.H. (2004). Two new compounds from the leaves of *Calocedrus microlepic* var. *formosana*. Chem. Pharm. Bull..

[43] Joseph-Nathan P., Santillan R.L., Gutierrez A. (1984). ^13^C-NMR study of cedrol, 6-isocedrol, and α-cedrene. J. Nat. Prod..

[44] Muto N., Tomokuni T., Haramoto M., Tatemoto H., Nakanishi T., Inatomi Y., Murata H., Inada A. (2008). Isolation of apoptosis-and differentiation-inducing substances toward human promyelocytic leukemia HL-60 cells from leaves of *Juniperus taxifolia*. Biosci. Biotechnol. Biochem..

[45] Smith E.C.J., Williamson E.M., Wareham N., Kaatz G.W., Gibbons S. (2007). Antibacterials and modulators of bacterial resistance from the immature cones of *Chamaecyparis lawsoniana*. Phytochemistry.

[46] Wong K.C., Sivasothy Y., Boey P.L., Osman H., Sulaiman B. (2010). Essential oils of *Etlingera elatior* (Jack) RM Smith and *Elingera littoralis* (Koenig) Giseke. J. Essent. Oil Res..

[47] Espinel-Ingroff A., Fothergill A., Ghannoum M., Manavathu E., Ostrosky-Zeichner L., Pfaller M., Rinaldi M., Schell W., Walsh T. (2005). Quality control and reference guidelines for CLSI broth microdilution susceptibility method (M 38-A document) for amphotericin B, itraconazole, posaconazole, and voriconazole. J. Clin. Microbiol..

