# Supplementary material for: Plant Disease Control Efficacy of Platycladus orientalis and Its Antifungal Compounds
Source: Plants (Basel). 2021 Jul 21;10(8):1496. doi: 10.3390/plants10081496 (PMC8400505; doi:10.3390/plants10081496)
Supplement: Supplementary file 1 [file plants-10-01496-s001.zip › plants-1280439-supplementary.pdf]

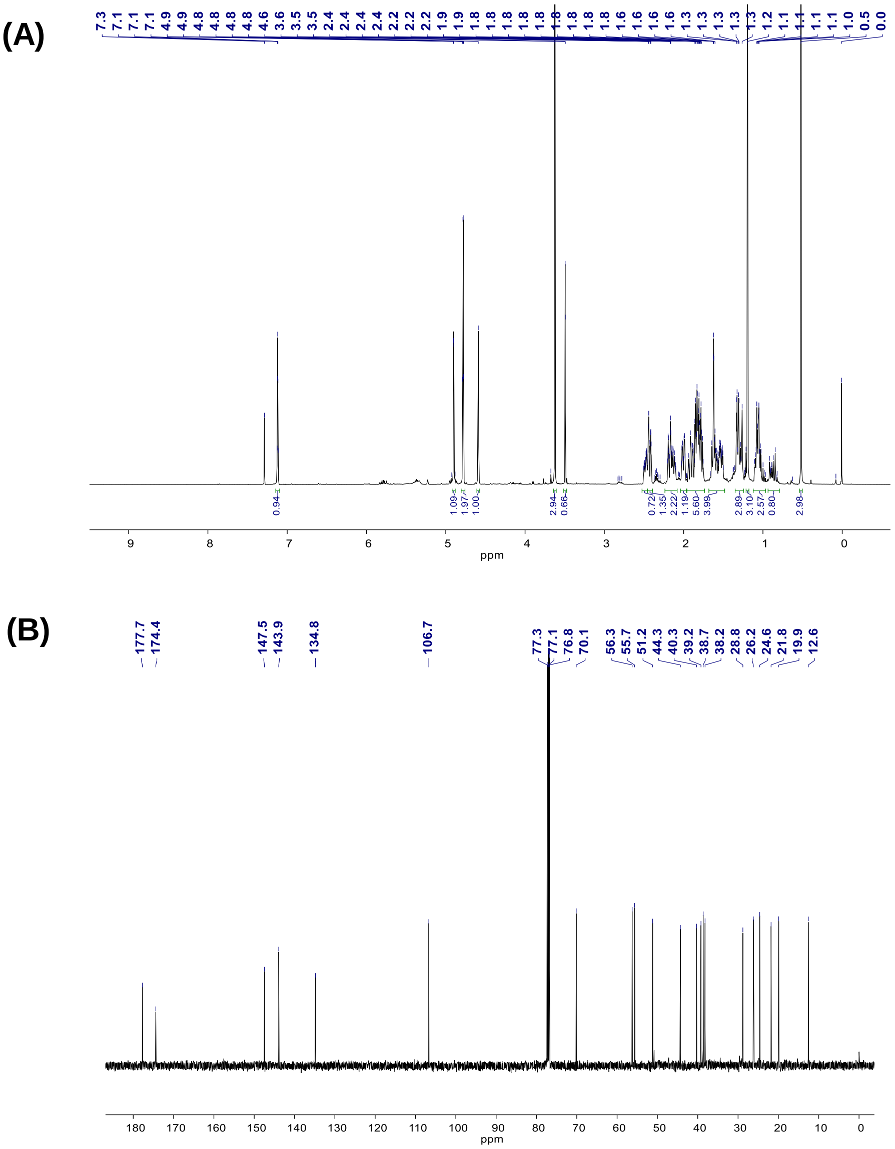

Figure S1. ^{1}H–NMR (A) and ^{13}C–NMR (B) spectra of pinusolide (compound **1)**.

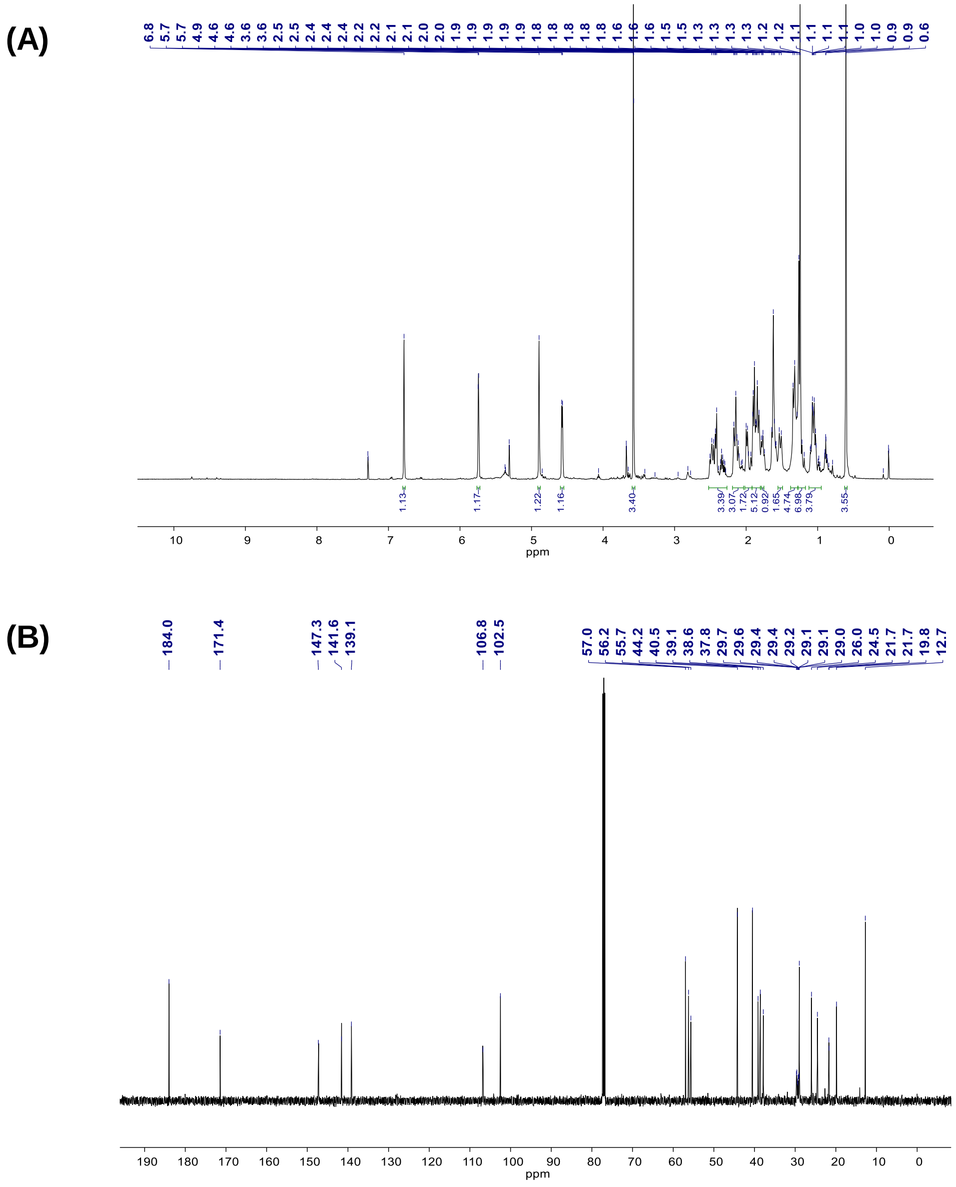

Figure S2. ^1H–NMR (A) and ^{13}C–NMR (B) spectra of 15-methoxypinusolidic acid (compound **2)**.

(A)

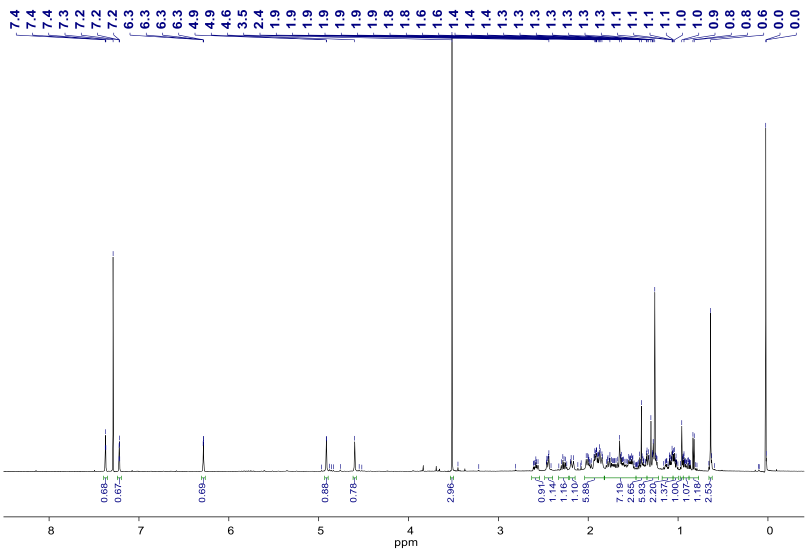

(B)

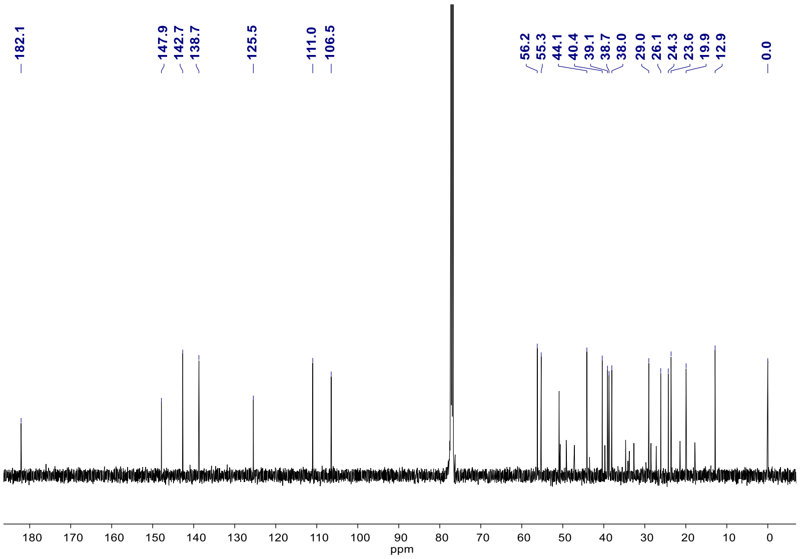

Figure S3. ^{1}H–NMR(A) and ^{13}C–NMR(B) spectra of lambertianic acid (compound **3)**.

(A)

(B)

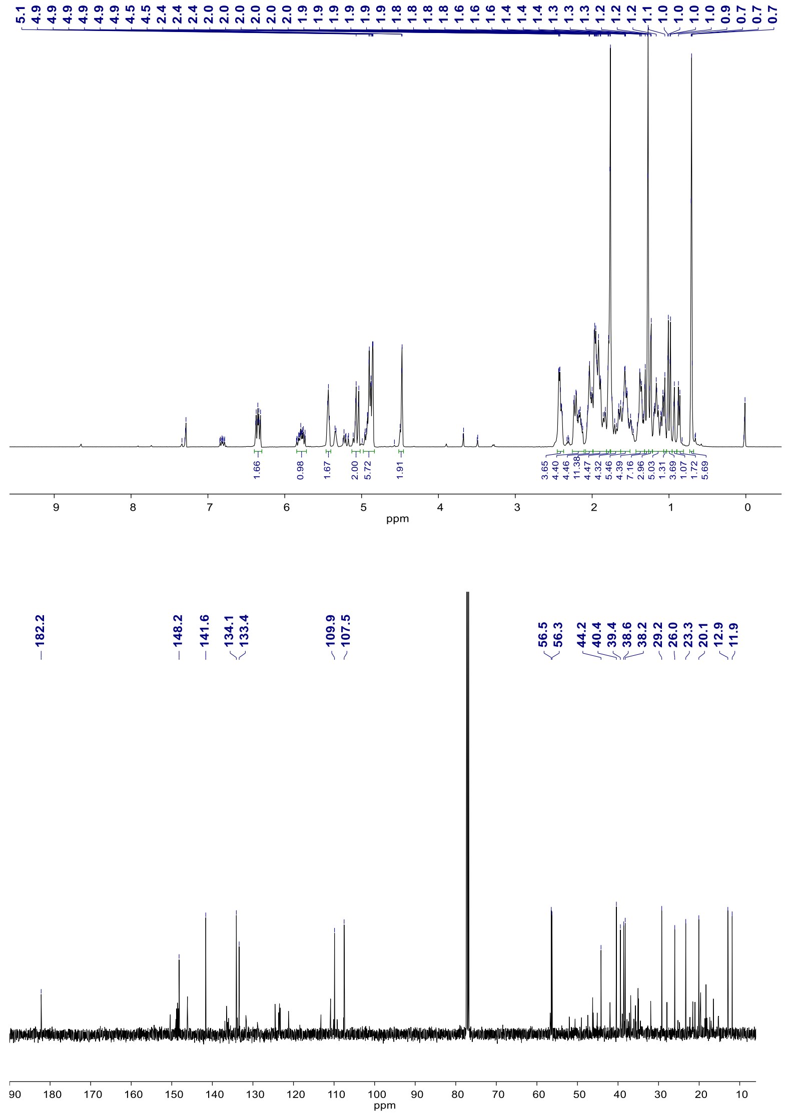

Figure S4. ^{1}H–NMR (A) and ^{13}C–NMR (B) spectra of *trans*-communic acid (compound **4)**.

(A)

(B)

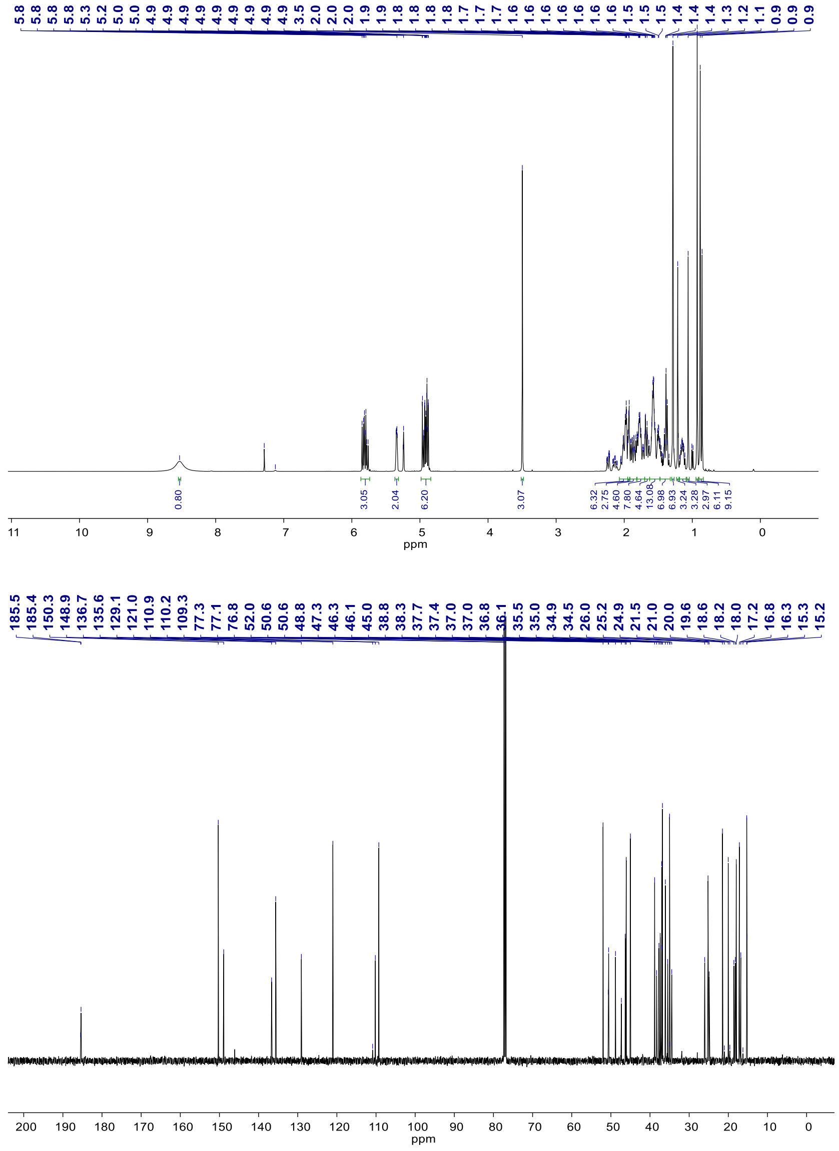

Figure S5. ^{1}H–NMR (A) and ^{13}C–NMR (B) spectra of the mixture of sandracopimaric acid (compound **5**) and isopimaric acid (compound **6**).

(A)

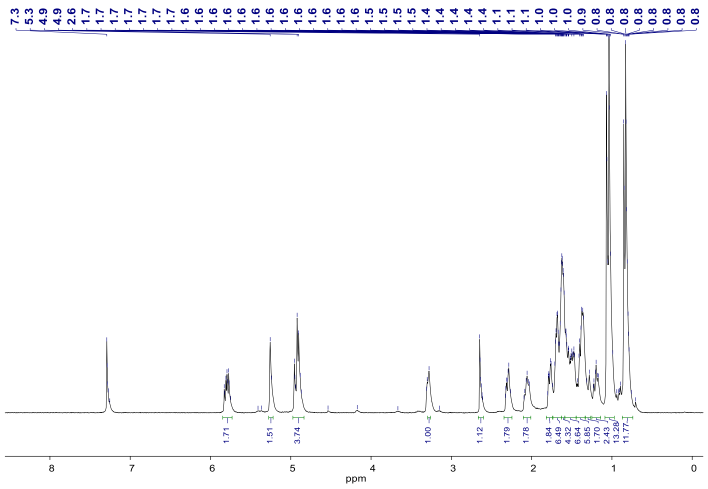

(B)

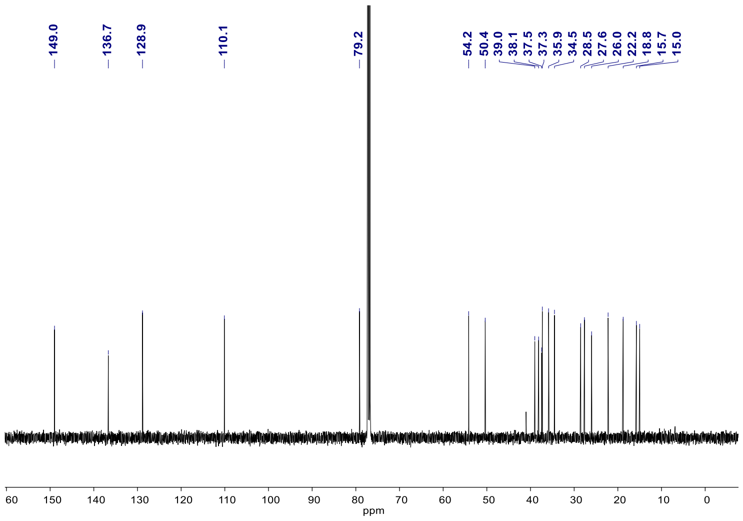

Figure S6. ^{1}H–NMR (A) and ^{13}C–NMR (B) spectra of sandaracopimaradien-3β-ol (compound **7**).

(A)

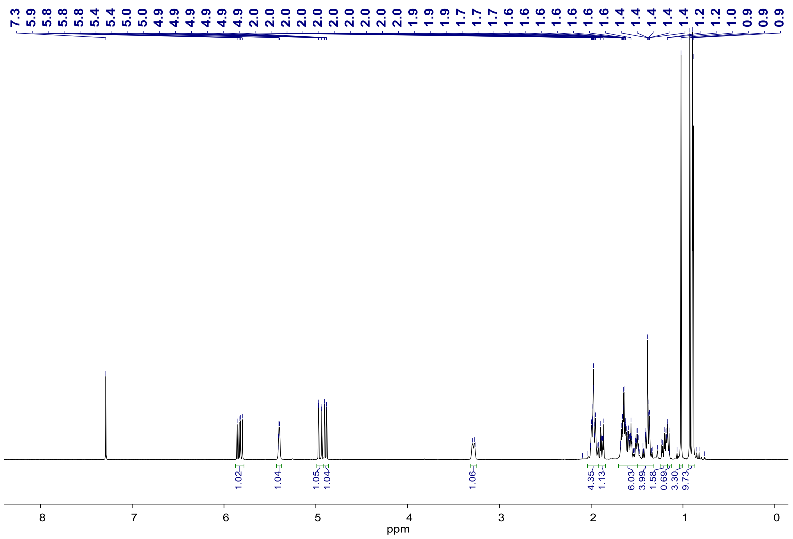

(B)

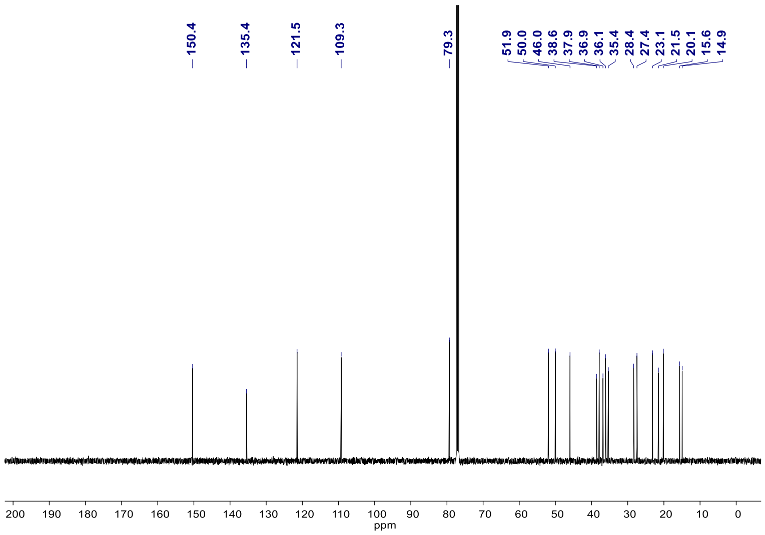

Figure S7. ^{1}H–NMR (A) and ^{13}C–NMR (B) spectra of isopimara-7,15-dien-3β-ol (compound **8**).

(A)

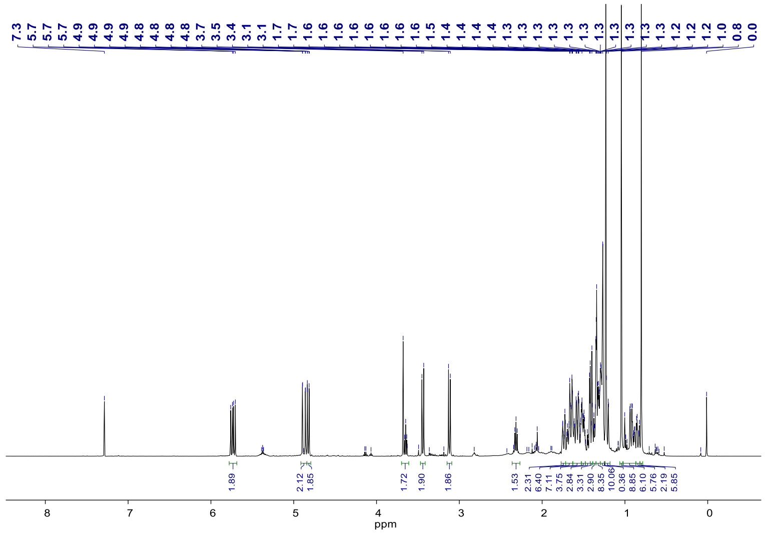

(B)

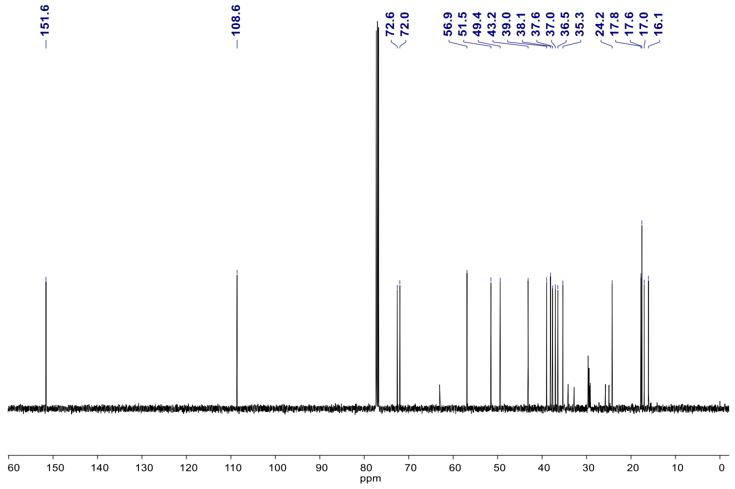

Figure S8. ^{1}H–NMR (A) and ^{13}C–NMR (B) spectra of 8β,18-dihydroxysandaracopimar-15-ene (compound **9**).

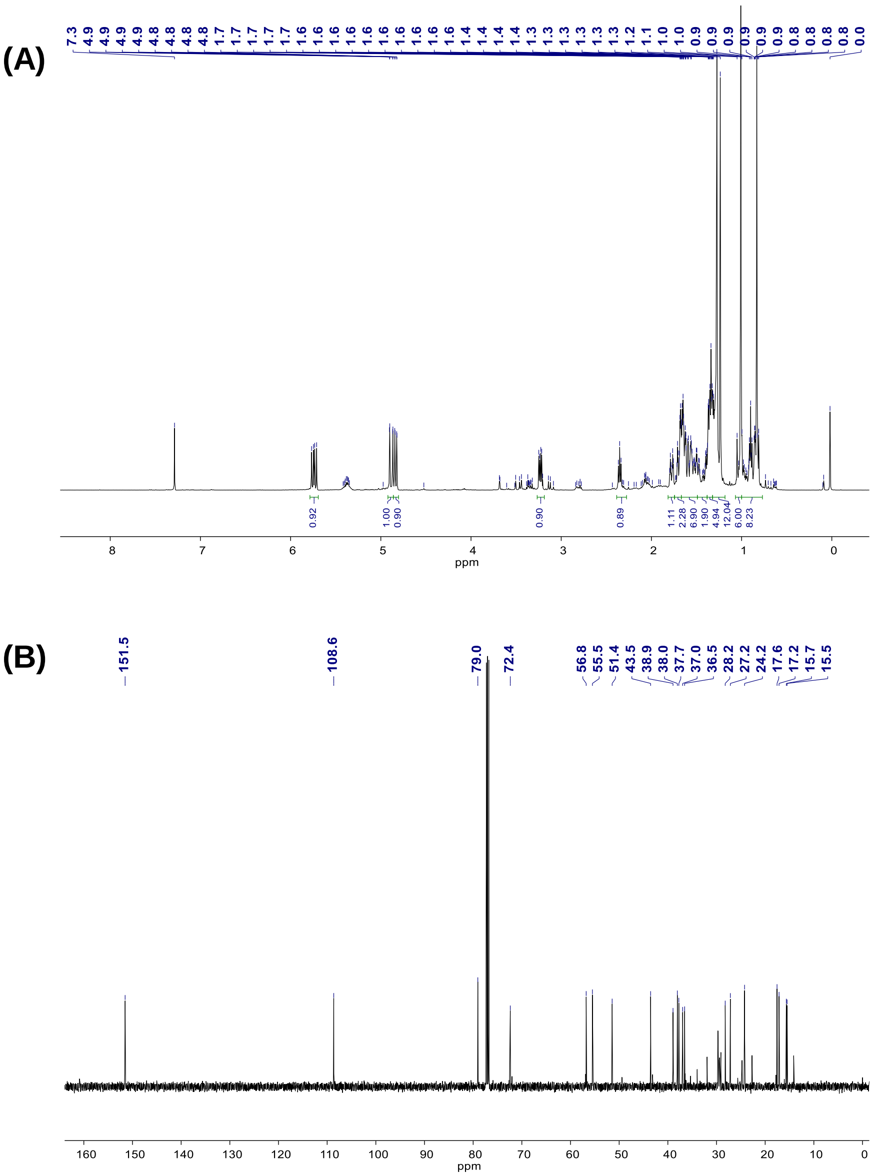

Figure S9. ^{1}H–NMR (A) and ^{13}C–NMR (B) spectra of 15-isopimaren-3β,8β-diol (compound **10**).

(A)

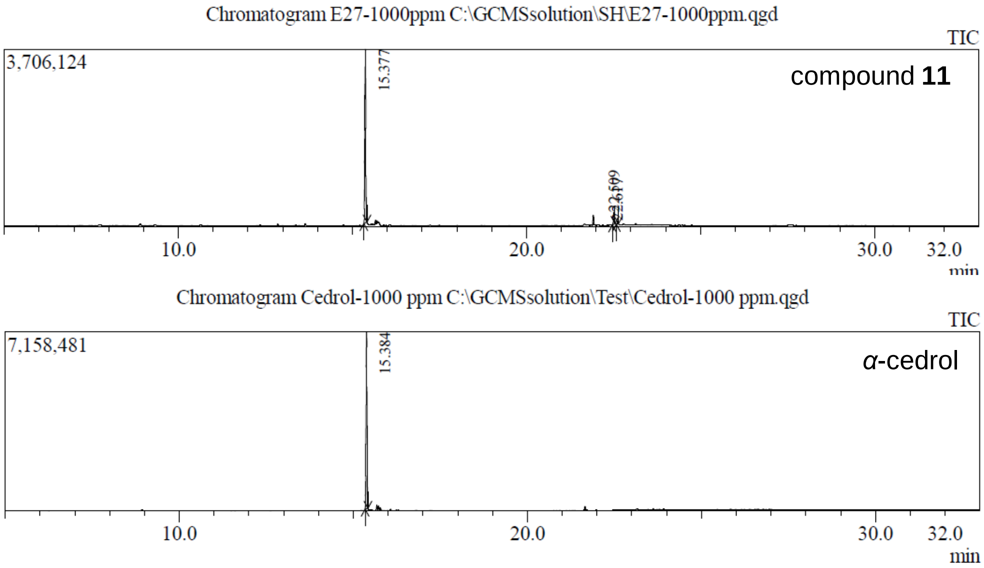

(B)

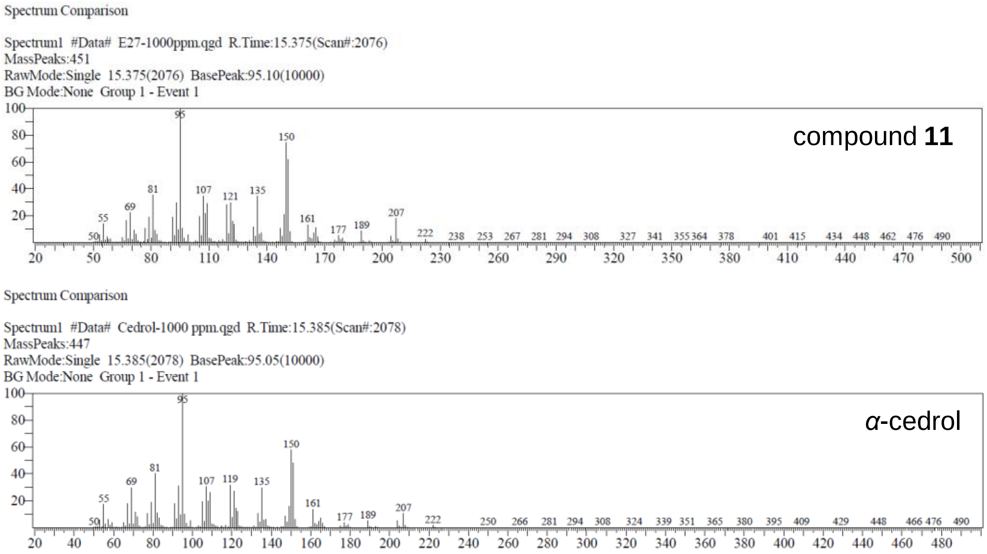

Figure S10. GC/MS chromatogram (A) and mass spectra (B) of compound **11** and *α*-cedrol, respectively. An authentic *α*-cedrol purchased from Sigma-Aldrich was used for this analysis.

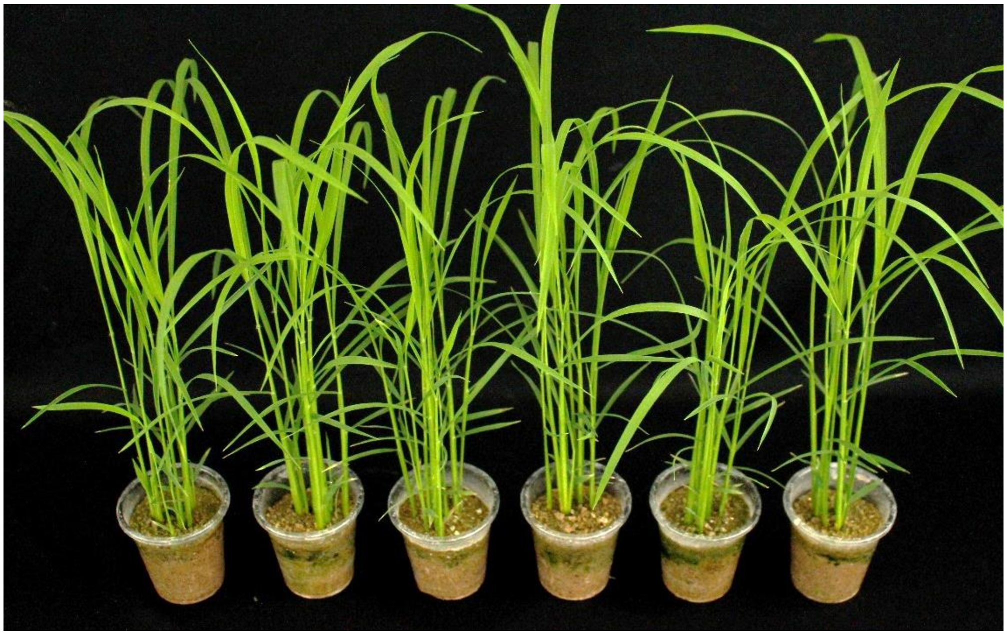

Figure S11. Plant treated with the pure compounds for phytotoxicity. Photos were taken 1 day after treatment of each compound.

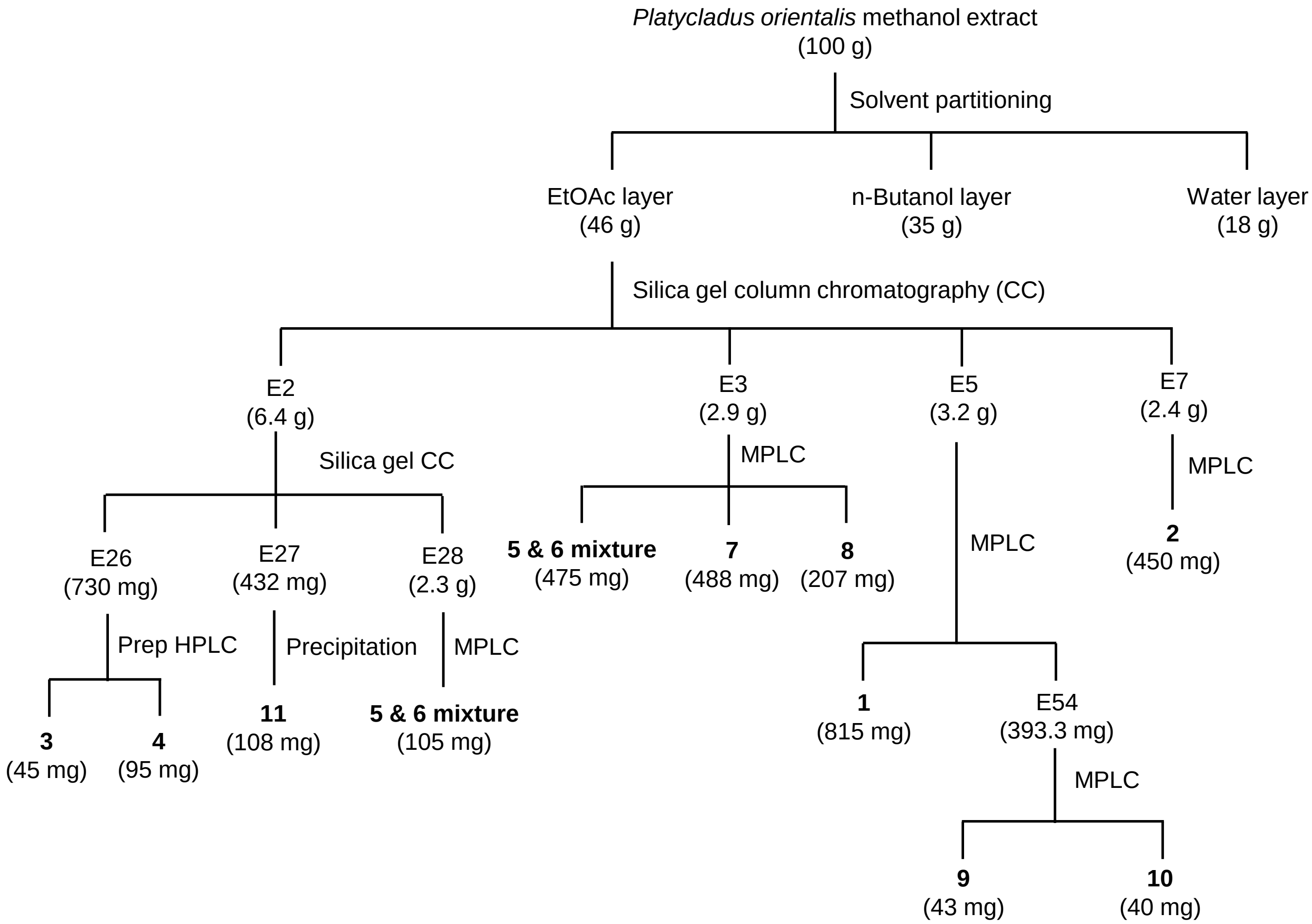

Figure S12. Isolation scheme of compounds 1-11 from *Platycladus orientalis.*

Table S1. MIC (μg/mL) values against *Magnaporthe oryzae* according to the 1:1 mixing ratio of pure compounds

	Compound **1**	Compound **2**	Compound **9**	Compound **11**
Compound **1**	–	–	–	–
Compound **2**	200	–	–	–
Compound **9**	100	200	–	–
Compound **11**	100	200	200	–

Table S2. Inoculation and incubation method for plant disease.

Plant disease	Developmental condition
Tomato late blight (TLB)	For TLB, two-leaf stages of tomato plants were inoculated by spraying with a zoospore suspension (2 × 10^4 sporangia/mL) of *Phytophthora infestans*. The inoculated plants were incubated in a humidified chamber (20 °C) for 2 days, and then the plants were transferred to a growth chamber (20 °C) for 1 day of incubation.
Tomato gray mold (TGM)	For TGM, two-leaf stage of tomato plants was inoculated by spraying with a conidial suspension (5 × 10^5 spores/mL) of *Botrytis cinerea*, and then the inoculated plants were incubated in a humidified chamber (20 °C) for 3 days.
Wheat leaf rust (WLR)	For WLR, one-leaf stage of wheat plants was sprayed with a spore suspension (0.67 g urediospores/L) collected from diseased wheat infected by *Puccinia triticina*. The inoculated plants were incubated in a humidified chamber (20 °C) for 1 day, and then the plants were moved to a growth chamber (20 °C) for 6 days of incubation.
Barley powdery mildew (BPM)	For BPM, a fully expended first leaf stage of barley plants were dusted with *Blumeria graminis* f. sp. *hordei* conidia formed on the leaves of barley. The inoculated plants were incubated in a growth chamber (20 °C) for 7 days.
Pepper anthracnose (PAN)	For PAN, a fully expanded two-leaf stages of pepper plants were inoculated by spraying with a spore suspension (5 × 10^5 spores/mL) of *Colletotrichum coccodes*. The inoculated plants were incubated in a humidified chamber (25 °C) for 2 days, and then the plants were transferred to a growth chamber (25 °C) for 1 day of incubation.

[Reference] Ngo et al., Antifungal properties of natural products from *Pterocarya tonkinensis* against phytopathogenic fungi. *Pest Manag. Sci.* **2021**, *77*, 1864-1872.
